# Isolation of quercetin and mandelic acid from *Aesculus indica* fruit and their biological activities

**DOI:** 10.1186/s12858-018-0095-7

**Published:** 2018-06-26

**Authors:** Muhammad Zahoor, Sadaf Shafiq, Habib Ullah, Abdul Sadiq, Farhat Ullah

**Affiliations:** Department of Chemistry, University of Malakand, Chakdara, Dir Lower KPK Pakistan

**Keywords:** *Aesculus indica*, HPLC, Quercetin, Mandelic acid

## Abstract

**Background:**

In this study *Aesculus indica* fruit was subjected to isolation of phytochemicals. Two antioxidants quercetin and Mandelic acid were isolated in pure state. The free radical scavenging and acetyl choline esterase inhibitory potential of the crude extract and sub fractions were also determined.

**Results:**

The antioxidant capacity of crude extract, fractions and isolated compounds were determined by DPPH and ABTS methods. Folin-Ciocalteu reagent method was used to estimate the total phenolic contents and were found to be 78.34 ± 0.96, 44.16 ± 1.05, 65.45 ± 1.29, 37.85 ± 1.44 and 50.23 ± 2.431 (mg/g of gallic acid) in crude extract, ethyl acetate, chloroform, n-hexane and aqueous fractions respectively. The flavonoid concentration in crude extract, ethyl acetate, chloroform, n-hexane and aqueous fraction were; 85.30 ± 1.20, 53.80 ± 1.07, 77.50 ± 1.12, 26.30 ± 1.35 and 37.78 ± 1.25 (mg/g of quercetin) respectively. The chloroform fraction was more potent against enzymes, acetyl choline esterase and butyryl choline esterase (IC_50_ = 85 and 160 μg/ml respectively). The phenolic compounds in the crude extract and fractions were determined using HPLC standard method. Chlorogenic acid, quercetin, phloroglucinol, rutin, mandelic acid and hydroxy benzoic acid were detected at retention times 6.005, 10.062, 22.623, 30.597, 35.490 and 36.211 in crude extract and different fractions. The ethyl acetate fraction was rich in the targeted compounds and was therefore subjected to column isolation. The HPLC chromatogram of isolated compounds showed single peak at specified retention times which confirms their isolation in pure state. The isolated compounds were then characterized by FTIR and NMR spectrophotometric techniques.

**Conclusion:**

The *Aesculus indica* fruit extracts showed antioxidant and anticholine esterase inhibitory potentials. Two bioactive compounds were isolated in the pure form ethyl acetate fraction. From results it was concluded that the fruit of this plant could be used to minimize oxidative stress caused by reactive oxygen species.

**Electronic supplementary material:**

The online version of this article (10.1186/s12858-018-0095-7) contains supplementary material, which is available to authorized users.

## Background

Plants have been remained the main source of medication for humans since time immorial due to their easy availability and low cost. The humans also uses medicinal plants to treat various diseases in animals as well [1, 2]. According to a survey conducted by W.H.O (World Health Organization) about 80% of world population uses herbal medication [3]. The use of medicinal plants for the treatment of various diseases in more prevalent in the developing countries About 50,000 out of 422,000 flowering plants have been documented to have potential pharmacological effects [4]. Initially these plants were used in crude form as drugs (powders, tinctures, in form of tea etc.) by humans [5]. The successful isolation of primary drugs like quinine, cocaine and codeine from plants opened a new era of herbal medication. Further work in this field led to the isolation of morphine from opium in the early 19th century, which was a milestone in herbal medication [6, 7].

The oxygen we take in respiration is converted into water. However sometime it is partially oxidized into a number of products collectively known as reactive oxygen species which are free radicals and they harm cells and biologically important substances like DNA and proteins resulting in a number of complication like aging, cancer etc. A substance which inhibits oxidation is known as *anti-oxidant*. The present interest in the analysis and use of antioxidant from medicinal plants is due to its wide use in scientific research and in industries [5–9]. Antioxidants maintain health and serve to decrease the levels of free radicals [9]. Many synthetic antioxidants are added to food as preservative in food industries. Some of them have shown toxic properties and are carcinogens. Therefore the scientists are forced to discover new antioxidants of nontoxic nature. Amongst the many sources plant are considered the most suitable and nontoxic [10].

*Aesculus indica* is member of Sapindaceae family. It is herbaceous plant which is commonly known as Jawaz [11] and found in temperate regions of Asia (Nepal, India, Pakistan and Afghanistan) at an altitude ranging from 900 to 3600 m [12]. Jawaz trees can attain an average height of 22.5 m. They shed leaves in winter. In the months of May and June these trees are in full bloom and yield multi colored buds. These buds on fertilization yield huge amount of seeds, which in the hilly area like Kashmir are consumed Kashmir stag [13].

*Aesculus indica* has remarkable medicinal properties. For the treatment of diabetes the whole plant is beneficial. Its fruit is also used in colic disorder. It is highly effective in complaints associated with veins for instance phlebitis, hemorrhoids, varicose veins and in ulcers to prevent thrombosis. Moreover also it also helps in fighting against some types of migraine and effusions of blood. Further it helps in limb complaints and frost bite. Its seed are also used as astringent and nutritious. The oil obtained from its seeds is used as cure for many skin diseases and also gives relief in rheumatism. Hitherto researchers have successfully isolated about 210 chemical compounds from Aesculus. Compounds like triterpenoid, triterpenoid glycosides (saponins), flavonoids, coumarins, carotenoid, long fatty chain compounds are only a few to name amongst them. In literature it is reported that *Aesculus indica* contain a mixture of saponins. One of these saponins is known as Aescine which has the ability of crystal formation. The aesculine obtained from this plant is used medicinally. The hydroxycoumarin glycoside aesculine reported from this plant, absorbs ultra violet rays at the bark of the branch [11–15].

From the last two decades the seeds and fruits of Aesculus have remained as major target for chemical investigators to isolate compounds from it [14]. Owing to the importance of natural antioxidants and *Aesculus indica*, this study was aimed to evaluate *Aesculus indica* for phenolic and flavonoid contents. The antioxidant and acetyl choline esterase inhibitory potential were also determined. Two natural antioxidants, quercetin and Mandelic acid were isolated from this plant and characterized through HPLC, FTIR and NMR.

## Results and discussion

The present investigation has led to the isolation of two reported compounds that have been isolated for the first time from *Aesculus indica* fruit. The structures and identification of isolated compounds were carried out with the help of different techniques FT-IR, ^1^H-NMR and HPLC. The biological activities of the isolated compounds and different fractions of crude extracts of the *Aesculus indica* fruit were also determined.

### DPPH radical scavenging effect

The antioxidant potential of crude extract and sub-fractions (ethyl acetate n-hexane, chloroform and aqueous fractions) were studied using DPPH and ABTS assays (concentration ranging from 1000, 500, 250, 125 and 62.5 μg/ml). The DPPH antioxidant assay is based on the ability of an antioxidant to scavenge DPPH free radical. The antioxidant donate electrons to DPPH free radical which results in color changes from purple to yellow. The percent free radical scavenging potential were found to be higher for chloroform fraction with IC_50_ value of 50 μg/ml followed by crude extract (IC_50_ = 60 μg/ml) (Table 1 and Additional file 1: Figure S1). The IC_50_ values of ethyl acetate; aqueous and n-hexane fractions were; 155, 250 and 725 μg/ml respectively. Ascorbic acid was used as positive control and its IC_50_ value was 15 μg/ml.

**Table 1:** Percent DPPH and ABTS radical scavenging potential of crude extract and their sub fractions of *Aesculus indica* fruit using ascorbic acid as standard

Samples	Concentrations (μg/mL)	ABTS percent inhibition (mean ± S.E.M)	ABTS IC_50_ (μg/mL)	DPPH percent inhibition (mean ± S.E.M)	DPPH IC_50_ (μg/mL)
Crude	1000	85.58 ± 1.31 ns		90.28 ± 0.50 ns	
	500	72.26 ± 1.18^***^		79.21 ± 0.48^***^	
	250	68.59 ± 1.00^***^	105	70.01 ± 0.80^***^	60
	125	54.93 ± 1.49^***^		58.23 ± 1.13^***^	
	62.5	42.66 ± 2.25^***^		50.45 ± 0.52^***^	
Chloroform	1000	83.39 ± 0.46^*^		84.27 ± 0.54^***^	
	500	72.58 ± 0.54^***^		73.66 ± 1.79^***^	
	250	63.30 ± 0.67^***^	65	64.56 ± 2.23^***^	50
	125	56.25. ± 2.14^***^		59.24 ± 0.69^***^	
	62.5	51.83 ± 0.84^***^		47.36 ± 0.76^***^	
Ethyl acetate	1000	78.31 ± 0.53^***^		79.04 ± 0.72^***^	
	500	66.99 ± 0.79^***^	183	68.77 ± 1.61^***^	
	250	56.29 ± 0.75^***^		57.33 ± 0.91^***^	155
	125	44.93 ± 0.88^***^		46.70 ± 1.22^***^	
	62.5	39.49 ± 0.63^***^		40.73 ± 0.37^***^	
Aqueous	1000	70.50 ± 0.74^***^		73.82 ± 1.20^***^	
	500	60.10 ± 2.29^***^	325	62.62 ± 1.16^***^	
	250	44.84 ± 2.67^***^		49.47. ± 0.58^***^	250
	125	35.76 ± 0.52^***^		36.56 ± 1.25^***^	
	62.5	20.66 ± 3.08^***^		26.16 ± 2.36^***^	
n-hexane	1000	58.13 ± 0.49^***^		57.03 ± 2.00^***^	
	500	43.17 ± 0.66^***^		44.44 ± 2.00^***^	
	250	31.01 ± 2.34^***^	760	35.22 ± 1.06^***^	725
	125	22.02 ± 1.11^***^		24.29 ± 0.54^***^	
	62.5	14.19 ± 0.55^***^		15.19 ± 0.50^***^	
Ascorbic acid	1000	89.94 ± 0.86		94.35 ± 0.7	
	500	86.29 ± 0.79		87.90 ± 0.96	
	250	77.32 ± 2.23	23	81.78 ± 2.16	15
	125	69.97 ± 3.26		73.61 ± 0.85	
	62.5	61.28 ± 1.31		63.31 ± 0.76	

Ascorbic acid was used as a positive control. Data is represented as mean ± S.E.M; (*n* = 3). Values significantly different as compared to positive control,^*^ *P* < 0.05; ^***^ *P* < 0.001, ns: *P* > 0.05

### ABTS radical scavenging effect

Chloroform fraction of *Aesculus indica* showed highest activity against ABTS free radical at various concentrations ranging from 1000, 500, 250, 125 and 62.5 μg/ml (IC_50_ = 65 μg/ml). The ABTS free radical scavenging potential of chloroform fraction was comparable to that of DPPH free radical scavenging capacity (Table 1 and Additional file 1: Figure S2). The crude extract also showed good antioxidant activity with IC_50_ = 105 μg/ml, followed by ethyl acetate, chloroform, aqueous and n- hexane fractions with 1C_50_ of 183, 350, and 760 μg/ml respectively. The ABTS percent inhibitions were compared with the positive control ascorbic acid with IC_50_ value of 23 μg/ml.

### Acetyl cholinesterase inhibition assays

The hydrolysis of acetylcholine can be stopped if AChE in the brain is inhibited. A number of plants have been used traditionally to enhance and alleviate other cognitive function and symptoms associated with Alzheimer’s disease. The AChE percent inhibition and their 1C_50_ values against the crude extract of *Aesculus indica* and their sub fraction are given Table 2. The crude extract showed good percent inhibition with IC_50_ value of 145 μg/ml. Amongst the sub fractions the chloroform fraction showed excellent percent inhibition with IC_50_ = 85 μg/ml followed by ethyl acetate, aqueous and n-hexane fraction with the IC_50_ of 190, 350 and 760 μg/ml respectively (Additional file 1: Figure S3). Galanthamine was used as a positive control in this study.

**Table 2:** Percent AChE and BChE inhibition potentials of *Aesculus indica* fruit Crude extract and their sub fractions

Sample	Concentration (μg/ml)	Percent AChE (mean ± SEM)	AChE IC_50_ (μg/ml)	Percent BChE (mean ± SEM)	BChE IC_50_ (μg/ml)
Crude	1000	84.32 ± 1.22^***^		80.42 ± 0.62^***^	
	500	71.23 ± 1.40^***^	145	68.48 ± 3.13^***^	200
	250	58.07 ± 1.20^***^		52.54 ± 1.26^***^	
	125	49.37 ± 0.02^***^		45.70 ± 0.52^***^	
Chloroform	1000	86.21 ± 0.49^***^		82.56 ± 0.70^***^	
	500	74.24 ± 0.81^***^	85	70.62 ± 0.80^***^	160
	250	64.22 ± 0.67^***^		58.011 ± 2.68^***^	
	125	53.82 ± 0.41^***^		48.05 ± 0.99^***^	
Ethyl acetate	1000	75.23 ± 0.55^***^	190	69.12 ± 2.60^***^	210
	500	63.91 ± 0.84^***^		60.63 ± 1.77^***^	
	250	54.53 ± 0.81^***^		51.06 ± 0.37^**^	
	125	40.61 ± 2.30^***^		37.80 ± 0.60^***^	
Aqueous	1000	71.46 ± 0.71^***^		68.87 ± 1.90^***^	
	500	58.10 ± 1.36^***^	350	54.18 ± 2.80^***^	400
	250	45.14 ± 0.67^***^		38.60 ± 3.46^***^	
	125	32.71 ± 0.76^***^		26.11 ± 0.42^***^	
n-Hexane	1000	54.15 ± 0.52^***^		52.76 ± 1.64^***^	
	500	44.28 ± 2.21^***^	760	40.56 ± 0.88^***^	870
	250	34.04 ± 0.91^***^		28.49 ± 2.24^***^	
	125	25.23 ± 0.88^***^		21.39 ± 0.69^***^	
Galanthamine	1000	95.32 ± 0.88		92.50 ± 0.71	
	500	84.31 ± 0.55	48	80.66 ± 1.20	66
	250	72.31 ± 0.60		72.72 ± 0.72	
	125	64.38 ± 0.54		60.83 ± 0.69	

Galanthamine was used as a positive control. Data is represented as (mean ± S.E.M) *n* = 3. Values significantly different as compared to positive control, ^**^ *P* < 0.01; ^***^ *P* < 0.001

### Butyryl cholinesterase inhibition assay

The inhibition potentials of the crude extract and their sub fraction of *Aesculus indica* against BChE are shown in the Table 2 and graphically in Additional file 1: Figure S4. The crude extract showed highest potential against BChE with IC_50_ value of 200 μg/ml. Amongst the fractions the chloroform fraction showed promising percent inhibition with IC_50_ value of 160 μg/ml. The other fractions like ethyl acetate, aqueous and n-hexane showed moderate percent inhibition with IC_50_ value of 210, 400 and 870 μg/ml respectively. The AChE and BChE inhibition potential of *Aesculus indica* has not been studied by any researcher and is therefore innovative.

### Flavonoids and phenolic contents

The crude extract showed highest phenolic content (78.34 ± 0.96 mg GAE/g) followed by chloroform fraction (65.45 ± 1.29 mg GAE/g). The aqueous fraction ranked third with value of 50.23 ± 2.431 mg GAE/g while in ethyl acetate and n-hexane fractions moderate quantities were present (44.16 ± 1.05 and 37.85 ± 1.44 mg GAE/g of dry sample respectively). The results are shown graphically in Additional file 1: Figure S5 while in tabulated form in Table 3.

**Table 3:** Total phenolic and total flavonoid contents in *Aesculus indica* fruit’s crude extract and Their sub fractions

Samples	Total phenolic (mg GAE/g) of dry sample)	Total Flavonoid (mg QE/g of dry sample)
Crude	78.34 ± 0.96	85.30 ± 1.20
Chloroform	65.45 ± 1.29	77.50 ± 1.12
Ethyl acetate	44.16 ± 1.05	53.80 ± 1.07
n-hexane	37.85 ± 1.44	26.30 ± 1.35
aqueous	50.23 ± 2.431	37.78 ± 1.25

The crude extract was rich in flavonoid content (85.30 ± 1.20 mg QE/g of dry sample). Amongst the fractions the chloroform fraction was rich in flavonoid content (77.50 ± 1.12 mg QE/g of dry sample), followed by ethyl acetate (53.80 ± 1.07 mg QE/g of dry sample), aqueous (37.78 ± 1.25 mg QE/g of dry sample) and n-hexane (26.30 ± 1.35 mg QE/g of dry sample) respectively (Table 3 and Additional file 1: Figure S6).

### Linear correlation of total phenolic and flavonoid contents vs antioxidant and anticholinesterase activities

**Fig. 1:**
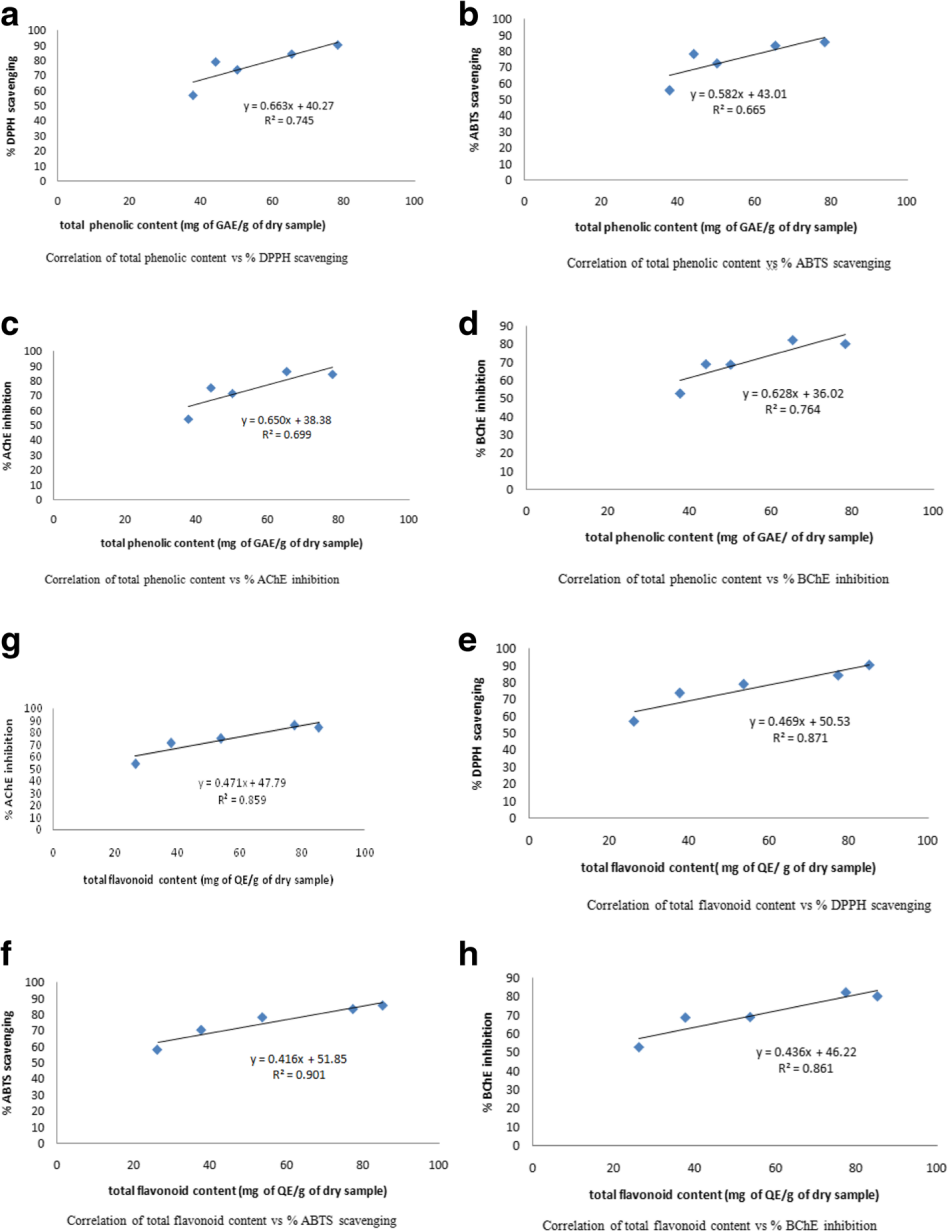
Linear correlations for total Phenolics Vs AChE (**c**), BChE (**d**), DPPH (**a**), and ABTS (**b**) and for total Flavonoid content Vs AChE (**g**), BChE (**h**), DPPH (**e**), and ABTS (**f**) activities

A linear correlation of total phenolic and flavonoid content vs various biological activities such as antioxidant (DPPH, ABTS) and Anticholinesterase (AChE, BChE) have been shown in the Fig. 1. The regression value of %AChE inhibition vs total phenolic content (Fig. 1c) and % BChE (Fig. 1d) are 0.669 and 0.764 respectively. Similarly % DPPH scavenging vs TPC (Fig. 1a) and % ABTS (Fig. 1b) are 0.745 and 0.665 respectively. The regression value of % AChE VS TFC (Fig. 1g) and % BChE (Fig. 1h) are 0.859 and 0.861 respectively. Also the % DPPH value vs TFC (Fig. 1e) and % ABTS (Fig. 1f) are 0.871 and 0.901 respectively. The good correlations have been observed between total flavonoid versus % DPPH and % ABTS. While moderate correlations have been established between % ABTS and % AChE vs total phenolic. From the regression values it was concluded that the antioxidant capacities exhibited by the extracts were due to the presence of high phenolic and flavonoid contents.

### Identification of phenolic compounds in *Aesculus indica* fruit through HPLC analysis

**Fig. 2:**
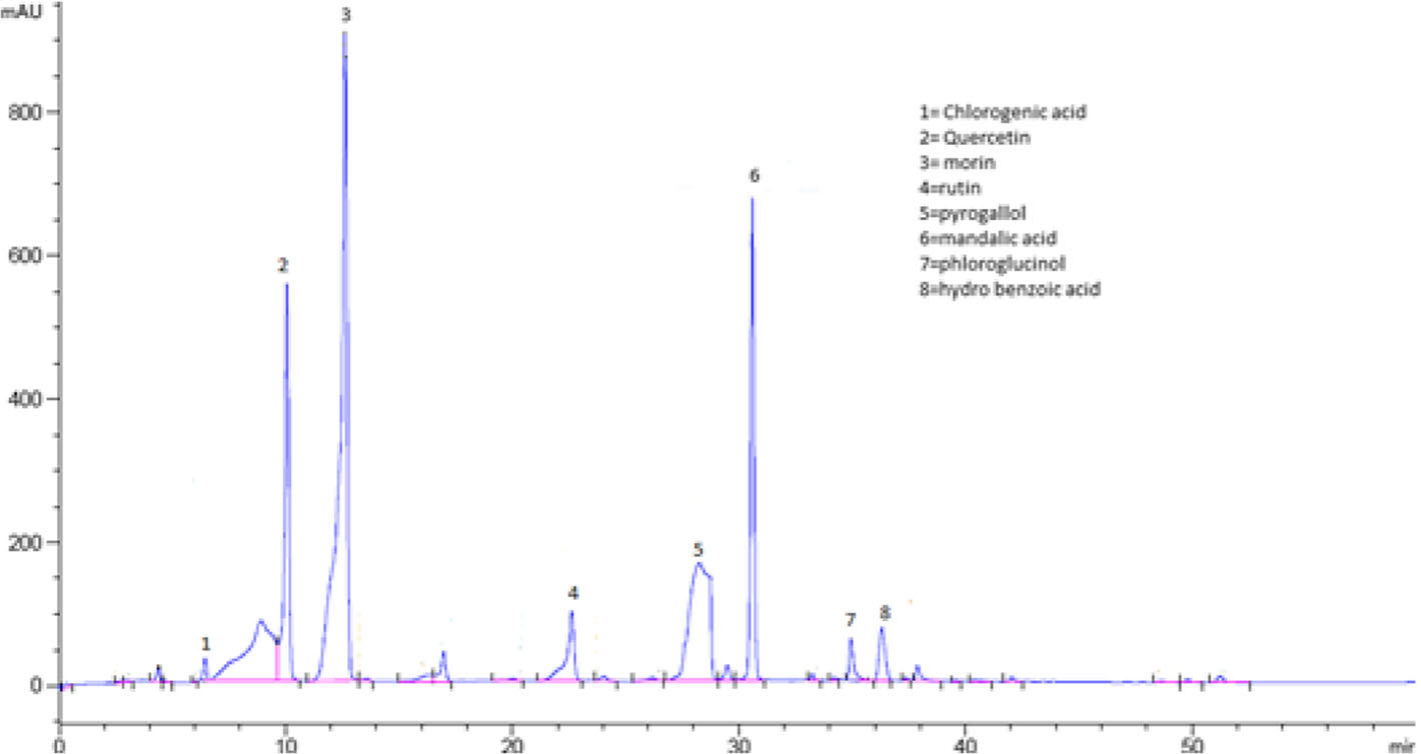
HPLC chromatogram of standards

**Fig. 3:**
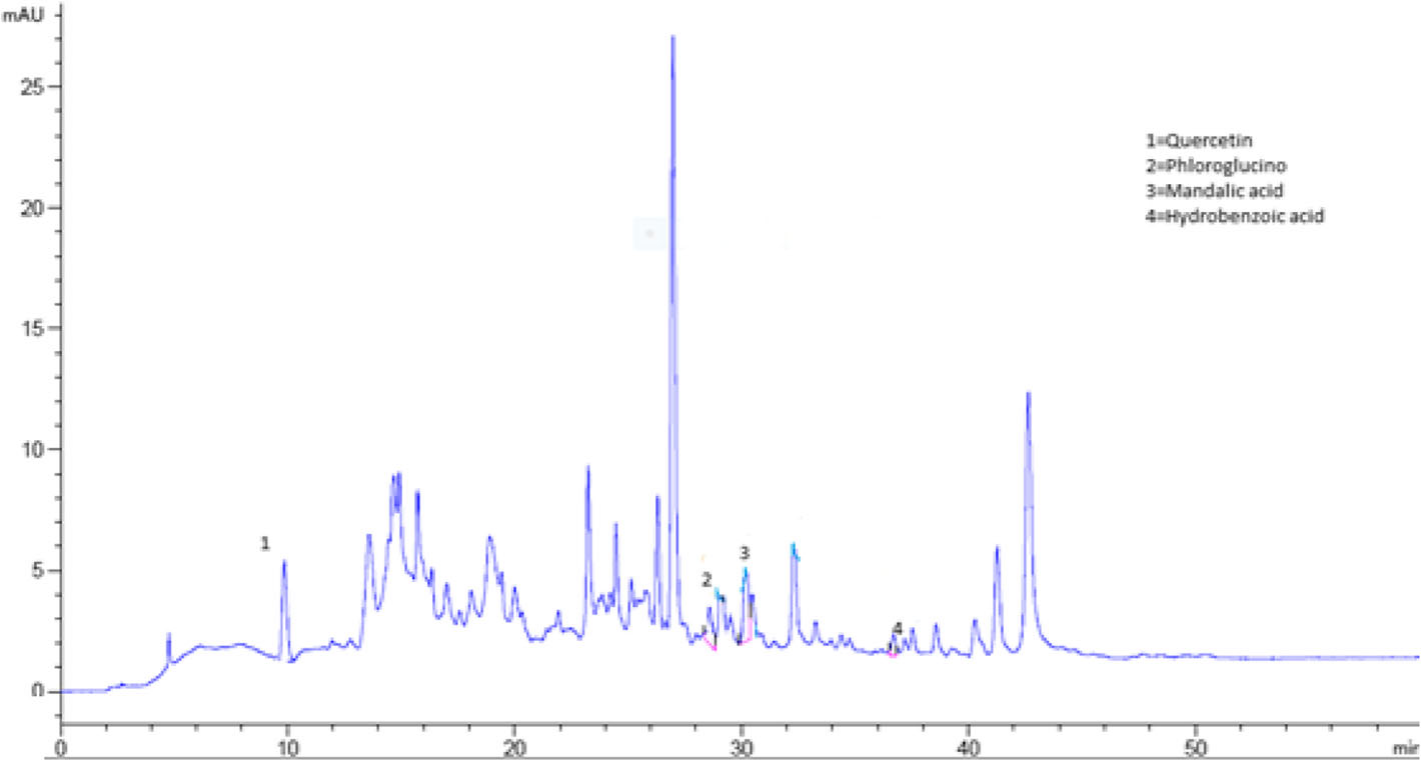
HPLC Chromatogram of crude extract

**Fig. 4:**
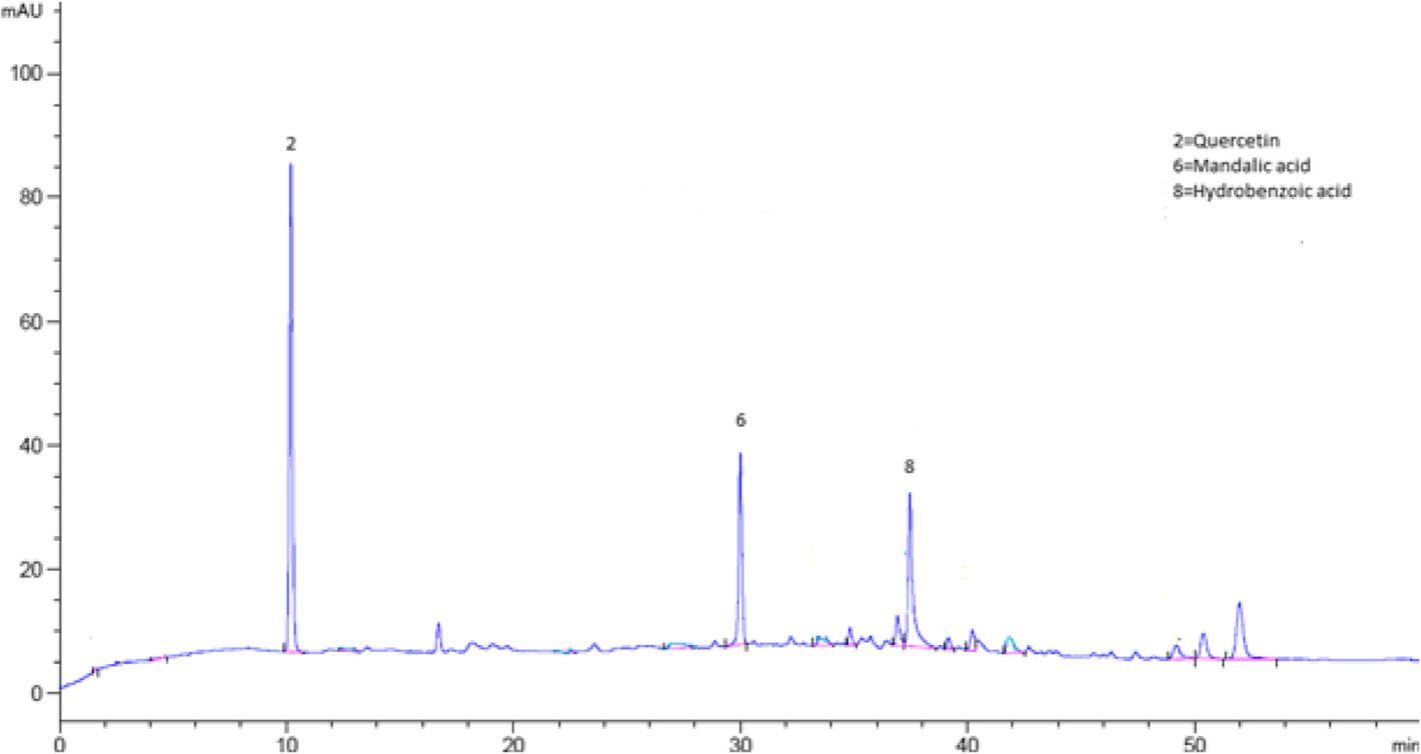
HPLC Chromatogram of Ethyl Acetate Fraction

**Fig. 5:**
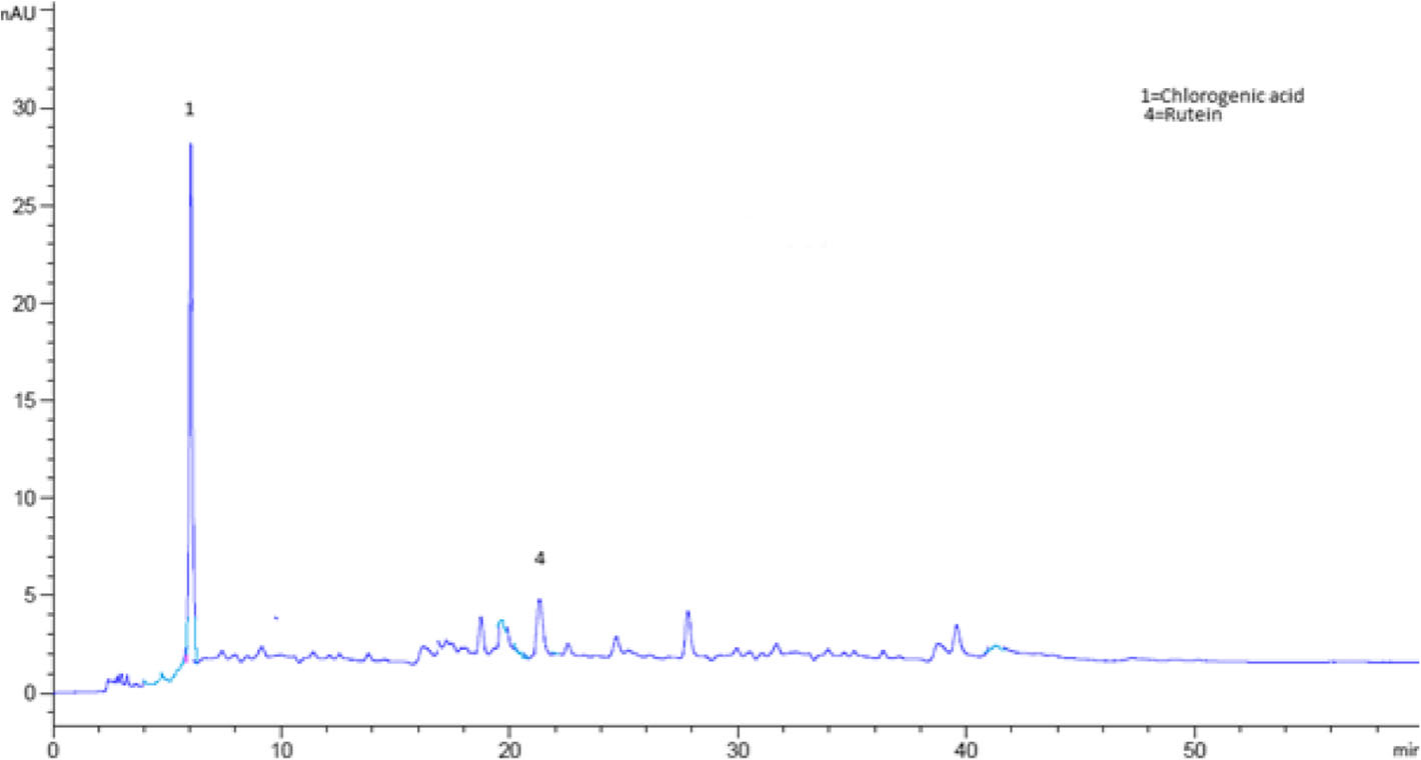
HPLC Chromatogram of Chloroform Fraction

**Fig. 6:**
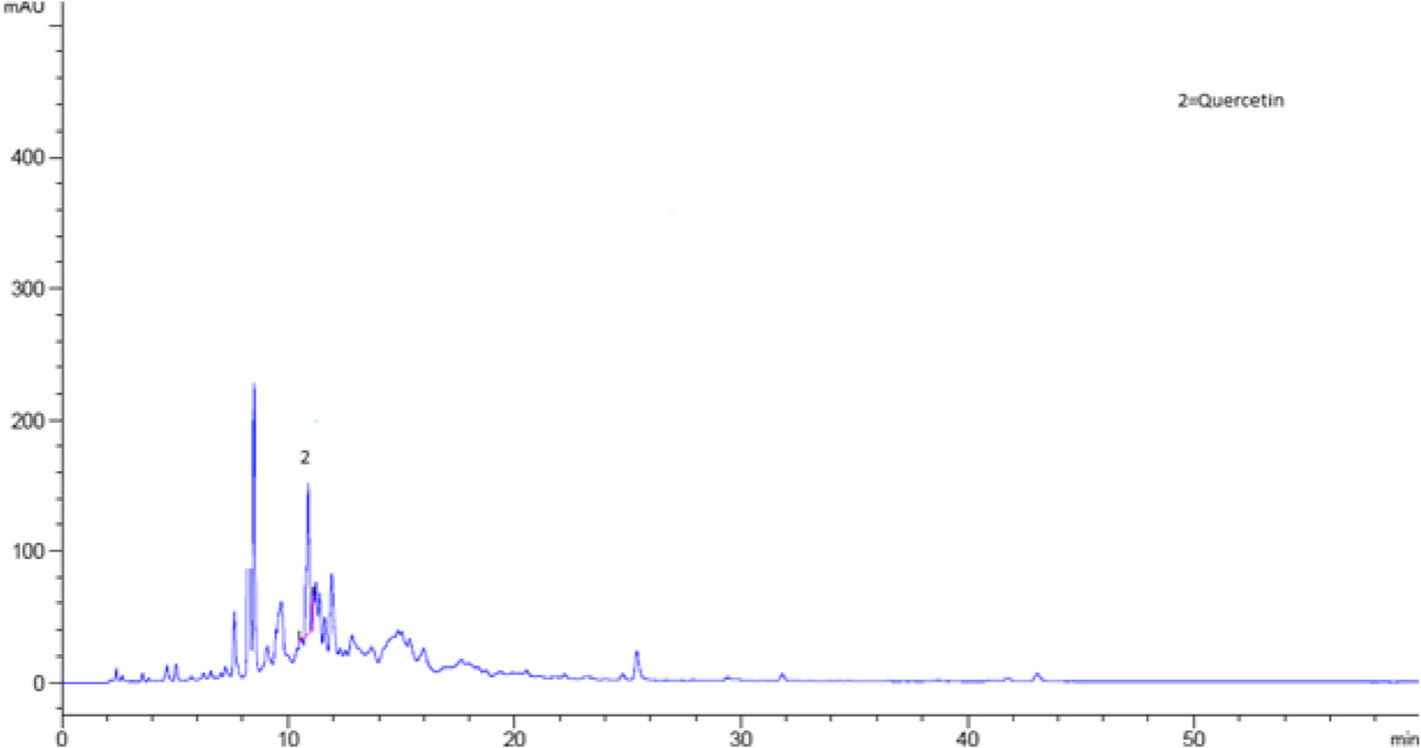
HPLC Chromatogram of n-hexane Fraction

**Fig. 7:**
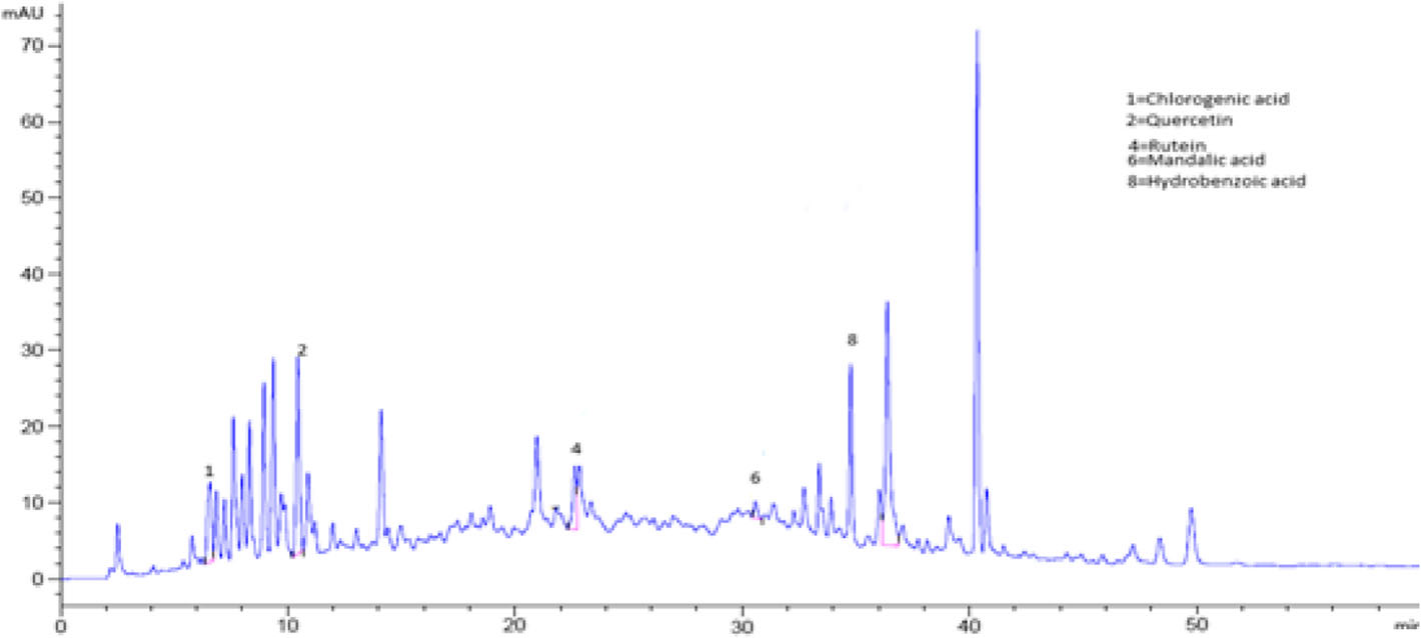
HPLC Chromatogram of Aqueous Fraction

The bioactive compounds present in the crude extract and fractions were identified by comparing their chromatograms with that of standards chromatogram (Fig. 2). In the crude extract only four phenolic compounds quercetin, phloroglucinol, mandelic acid and hydroxy benzoic acid were identified (Fig. 3). Among the four phenolic compounds quercetin was present in greater concentration followed by hydroxy benzoic acid, mandelic acid and phloroglucinol (Table 4). In ethyl acetate fraction only three phenolic compounds were detected i-e quercetin, mandelic acid and hydroxy benzoic acid (Fig. 4). Quercetin is present in greater concentration followed by hydroxy benzoic acid and mandelic acid (Table 5). In chloroform fraction only two phenolic compounds were detected i-e chlorogenic acid and rutin (Fig. 5). Chlorogenic acid is present in greater concentration (Table 6). In n-hexane fraction only one phenolic compound was detected, and that was quercetin (Fig. 6) whose concentration was very low (Table 7). In Aqueous fraction five phenolic compounds were detected viz. chlorogenic acid, quercetin, rutin, mandelic acid and hydroxy benzoic acid (Fig. 7). Hydroxy benzoic acid was present in greater amount while other phenolic compounds were present in less amount (Table 8).

**Table 4:** Concentration of antioxidants in crude extract

S. No	Peak	Retention time (min)	Possible identity	Quantity (mg/100 g)	Identification Reference
1	2	10.062	Quercetin	1.22	Standard
2	6	30.597	Mandelic acid	4.52	Standard
3	7	35.490	Phloroglucinol	1.53	Standard
4	8	36.211	Hydroxy benzoic acid	0.24	Standard

**Table 5:** Concentration of antioxidants in ethyl acetate fraction

S. No	Peak	Retention time (min)	Possible identity	Quantity (mg/100 g)	Identification reference
1	2	10.062	Quercetin	6.74	Standard
2	6	30.597	Mandelic acid	5.42	Standard
3	8	35.490	Hydroxy benzoic acid	0.965	Standard

**Table 6:** Concentration of antioxidants in chloroform fraction

S. No	Peak	Retention time (min)	Possible identity	Quantity (mg/100 g)	Identification reference
1	1	6.005	Chlorogenic acid	1.520	Standard
2	4	22.623	Rutin	3.67	Standard

**Table 7:** Concentration of antioxidants in n-hexane fraction

S. No	Peak	Retention time (min)	Possible identity	Quantity (mg/100 g)	Identification reference
1	2	10.062	Quercetin	0.0406	Standard

**Table 8:** Concentration of antioxidants in aqueous fraction

S. No	Peak	Retention time (min)	Possible identity	Quantity (mg/100 g)	Identification reference
1	1	6.005	Chlorogenic acid	1.617	Standard
2	2	10.062	Quercetin	3.92	Standard
3	4	22.623	Rutin	0.014	Standard
4	6	30.597	Mandelic acid	2.68	Standard
5	8	36.211	Hydroxy benzoic acid	3.19	Standard

### Isolation of phenolic compounds from *Aesculus indica*

The ethyl acetate fraction was evaporated and its slurry with silica gel was developed which was then subjected to column chromatography. The column was eluted successively, using n-hexane and ethyl acetate gradient that afforded various sub fractions with increasing order of polarities (1 to 60%). From the TLC analysis, fractions were combined according to their separation profile. Twelve sub fractions (C-1 to C-12) were obtained which were then subjected to pencil silica gel column for further purification. The purified isolated compounds were then subjected to HPLC analysis for purity check and NMR and FTIR for structural elucidation. The single peak in HPLC chromatogram (broad peak) confirms their purity and isolation.

#### Compound 2 (Quercetin)

**Fig. 8:**
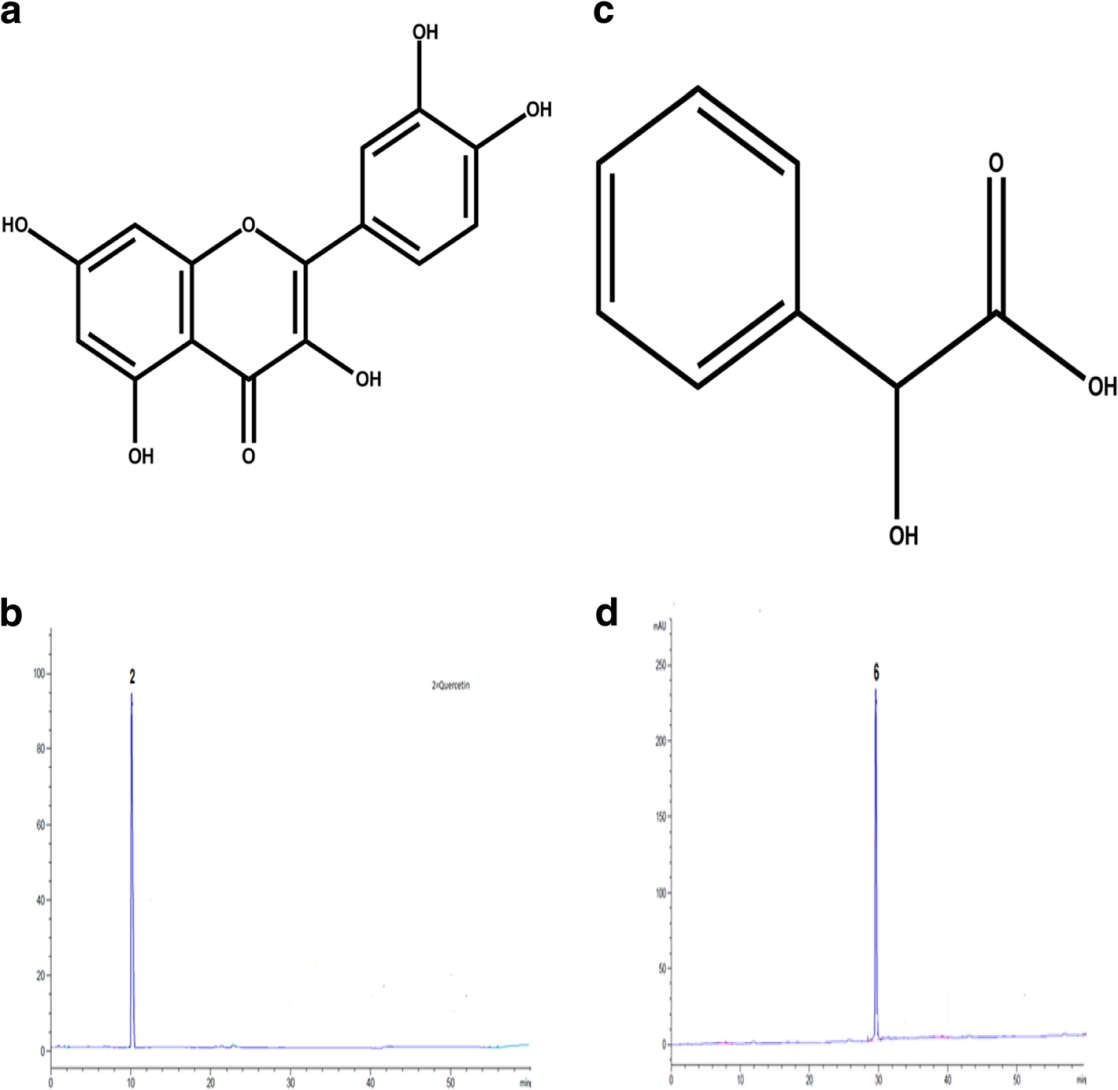
Structural formulae and HPLC chromatograms of isolated compounds (**a** = quercetin, **b** = HPLC chromatogram of quercetin, **c** = structure of mandelic acid **d** = HPLC chromatogram of mandelic acid)

The Quercetin; 2-(3,4-dihydroxy phenyl)-3,5,7-trihydroxy-4H-Chromen-4-one was isolated from the ethyl acetate fraction as yellow powder. Its molecular formula is C_15_H_10_O_7_ while its structural formula is given in Fig. 8. The HPLC analysis was carried out to confirm their purity and isolation (Fig. 8). The FTIR spectra of isolated quercetin is shown in Additional file 1: Figure S7. The absorption bands at 3520 cm^− 1^ representing hydroxyl group. The peak at 3050 cm^− 1^ is for C-H bond. The presence of aromatic nucleus is evident from aromatic C=C bond stretching at1600 cm^− 1^. In ^1^H-NMR (Additional file 1: Figure S8) spectral data (CD_3_OD) (Varian 300Mhz) the peaks at 6.28 (1H,CH), 6.48 (1H,CH), 6.98 (1H,CH), 7.63 (1H,CH) and 7.82(1H,CH) ppm show the aromatic proton at C-6, C-8, C-5, C-6 and C-2 respectively. The present data was in good correlation to the previously reported data by Selvaraj et al. [16].

#### Compound 6 (Mandelic acid)

The compound 6 mandelic acid (2-hydroxy-2-phenyle acetic acid) was isolated from the ethyl acetate fraction as white crystalline solid. Its molecular formula is C_8_H_8_O_3_ and its structural formula is shown in Fig. 8. The HPLC chromatogram was developed to confirm their purity and isolation (Fig. 8). The single broad peak in HPLC chromatogram confirms its isolation in the pure form. The FTIR spectra of isolated mandelic acid is shown in Additional file 1: Figure S9. The absorption broad band at 3400 cm^− 1^ indicating the presence of carboxyl group. The peak at 3050 cm^− 1^ representing C-H group while aromatic C=C bond stretching at 1600 cm^− 1^ shows the presence of the aromatic nucleus and the peak at 1710 cm^− 1^ shows the carbonyl functionality. The ^1^H-NMR spectra (Additional file 1: Figure S10) shows a singlet peak at 12.4 ppm indicating carboxylic proton and the signal at 5.2 ppm can be assigned to aliphatic proton at C-1 and C-2 respectively. The doublets signal at 7.6, 7.4 ppm indicating the aromatic proton at C-4 and C-5 respectively. In the same way the triplet signal appeared at 7.1 ppm which could be assigned to aromatic proton at C-6.

### Antioxidant potential of the isolated compounds

The phenolic compounds; quercetin and mandelic acid isolated from the fruit of *Aesculus indica* showed excellent antioxidant activity against DPPH and ABTS free radicals at different concentration ranging from 62.5–1000 μg/ml. The percent free radical scavenging potential was higher for quercetin with IC_50_ value of 78 μg/ml as compared to mandelic acid (IC_50_ = 118 μg/ml). The quercetin and mandelic acid also exhibited good antioxidant activity against free radical ABTS with IC_50_ values of 180 and 242 μg/ml respectively (Table 9 and Additional file 1: Figures S11 and S12). Ascorbic acid was used as a standard and its IC_50_ value was 38, 58 μg/ml for DPPH and ABTS respectively.

**Table 9:** Percent ABTS and DPPH Radical Scavenging Potential of quercetin and Mandelic acidisolated from Aesculus Indica Fruit

Samples	Concentrations (μg/mL)	DPPHpercent inhibition (mean ± S.E.M)	DPPHIC_50_ (μg/mL)	ABTS percent inhibition (mean ± S.E.M)	ABTSIC_50_ (μg/mL)
Quercetin	1000	81.91 ± 0.70^***^		79.80 ± 2.51^**^	
	500	70.75 ± 1.72^***^		66.08 ± 0.40^***^	
	250	58.25 ± 1.30^***^	78	55.01 ± 1.60^***^	180
	125	48.44 ± 0.62^***^		43.30 ± 3.13^***^	
	62.5	35.67 ± 0.59^***^		32.46 ± 0.50^***^	
Mandelic acid	1000	78.54 ± 2.00^***^		72.70 ± 1.54^***^	
	500	67.16 ± 0.56^***^		62.60 ± 1.72^***^	
	250	57.71 ± 0.52^***^	120	50.66 ± 2.20^***^	240
	125	45.50 ± 2.56^***^		39.84 ± 0.99^***^	
	62.5	30.57 ± 1.23^***^		27.15 ± 0.76^***^	
Ascorbic acid	1000	94.35 ± 0.73		89.94 ± 0.86	
	500	85.92 ± 0.96		80.29 ± 0.79	
	250	80.74 ± 1.19	38	70.22 ± 2.23	58
	125	67.61 ± 0.80		62.97 ± 2.26	
	62.5	56.31 ± 0.76		50.88 ± 0.30	

Ascorbic acid was used as a positive control. Data is represented as mean ± S.E.M; (n = 3). Values significantly different as compared to positive control, ^**^ *P* < 0.01; ^***^ *P* < 0.001

## Conclusion

In this study fruit of *Aesculus indica* was screened for phenolic compounds. The antioxidant potential of the crude extract, fractions and isolated compounds were determined through DPPH and ABTS assays. The crude extract and different fraction were also tested for their acetyl choline esterase inhibitory potential. The chloroform fraction was the most potent fraction against AChE and BChE. The phenolic and flavonoids contents were also determined. In the crude extract and sub fractions chlorogenic acid, quercetin, phloroglucinol, rutin, Mandelic acidand hydroxy benzoic acid were identified through reverse phase HPLC. The ethyl acetate fraction was found to be rich in phenolic active compounds and was therefore subjected to column isolation. Two compounds, quercetin and mandelic acid were isolated in the pure state. The isolation and purity of isolated compounds were evident from broad single peak in the HPLC chromatogram. The isolated compounds were then confirmed through FTIR and ^1^HNMR. From the results it was concluded that the fruit of this plant is a good radical scavenger which could be used to minimize oxidative stress caused by reactive oxygen species. Studies are needed in the same lines to evaluate other potentials of this plant as well.

## Methods

### Plant sample collection

The fruits of the *Aesculus indica* were collected from Laram Mountain village Ouch, Dir Lower KPK Pakistan in January 2015 and were authenticated by the Botanist in the department of Botany University of Malakand. A voucher specimen (1020HU) was also deposited in the Herbarium of Malakand University.

### Chemicals, drugs and standards

Quercetin, morin, rutin, pyrogallol, mandelic acid, hydroxy benzoic acid, phloroglucinol and chlorogenic acid, Follin-ciocalteu reagent, sodium carbonate, 2,2-diphenyle-1 picrylhydrazyle [DPPH], aluminum chloride, sodium nitrate, sodium hydroxide, ethanol, methanol, ascorbic acid, distilled water, 5,5-dithio-bis-nitrobenzoic acid (DTNB), 2,2′-azino-bis-3 ethylbenzothiazoline-6-sulfonic acid (ABTS) were all purchased from Sigma Aldrich. Butyrylthiocholine iodide, acetylthiocholine iodide, acetyl cholinesterase from Electric eel (type-VI-S) and butyrylcholinesterase from Equine were obtained from Sigma-Aldrich USA and were used in enzyme inhibition assays. All solvents used in the assays were of HPLC grades purchased from Dae-Jung Korea.

### Extraction and fractionation

Fresh fruits of the *Aesculus indica* were thoroughly washed with tape water to remove dust and soil contaminants. After this for drying it was kept at room temperature in a shady place for 4 weeks. The dried plant sample was grinded into fine powder. To prepare crude extract, the powder sample was soaked in 90% methanol for 72 h. Then the mixture was subjected to filtration through whattman filter paper and the residue left was again dipped in methanol for additional 72 h and the mixture was filtered again. The filtrates obtained from above two steps were then combined. The filtrates were then concentrated at 40 °C in rotary evaporator (Rota vapor R-200 Buchi, Switzerland) and the extract was obtained in semisolid form. It was then kept in open atmosphere to evaporate the remaining solvent and was converted into solid form. The crude extract was subjected to fractionation. In brief 300 g crude extract was suspended in 900 ml of methanol and subjected to solvent-solvent extraction for fractionation. All these fractions were separated in separating funnels and stored into flasks and labeled with different code and kept in a refrigerator at a temperature of 4 °C. All fractions collected were then subjected to isolation of antioxidants and biological activities.

### Determination of free radical scavenging activity using DPPH assay

The free radical scavenging ability of the extracts were determined by using 2, 2- Diphenyl-1-picrylhydrazyl (DPPH) assay modified by Brand-Williams [17]. DPPH solution was prepared by dissolving 20 mg in 100 ml of methanol (stock solution) and then from this solution 3 ml were taken and its absorbance at 515 nm was adjusted to 0.70. This was considered as control solution. This stock solution was then covered with aluminum foil and kept in dark place for 24 h for the formation of free radical. After this 5 mg was taken from each extracts and were dissolved in 5 ml of methanol (stock solutions). From each stock solution different diluted solutions such as 1000, 500, 250, 125, 62.5 μg/ml were prepared through serial dilution. From these diluted solutions, 2 ml were mixed with 2 ml DPPH and were allowed to react in darkness for 15 min. The following equation was used to calculate the percent inhibition of DPPH by extracts:

1 $$ \% inhibition=\frac{A-B}{A}\times 100 $$

Where; A = absorbance of pure DPPH in oxidized form.

B = absorbance of sample taken after 15 min of reaction with DPPH.

For the determination of IC_50_ values of the extracts the same concentrations solutions of ascorbic acid were prepared (1000, 500, 250, 125, 62.5 μg/ml) and same procedure was used to determined its percent DPPH free radical inhibition. The IC_50_ is the concentration at which 50% of DPPH solution is scavenged.

### Free radical scavenging activity by ABTS assay

The ABTS free radicals scavenging by crude extract and sub-fractions were determined following standard procedure of Re et al. [18]. For the preparation of ABTS free radical solution, 7 mM of ABTS and potassium per sulphate (2.45 mM) were dissolved separately in 100 ml of methanol and thoroughly mixed. To produce ABTS free radical, the mixture was kept in dark for one night. The absorbance of ABTS free radical was adjusted to 0.7 at 745 nm by dilution with 50% methanol. Three-hundred microliter of test samples (extracts) were mixed 3 ml of ABTS solution and were kept for 15 min in incubator at 25 °C. After incubation the absorbance of the mixture was measured at 745 nm using a double beam spectrophotometer. The same procedure was followed for the preparation of different dilution of ascorbic acid which was used as positive control. The percent ABTS free radical inhibition was calculated by formula:

2 $$ \mathrm{Percent}\ \mathrm{scavenging}\ \mathrm{activity}=\frac{A-B}{A}\times 100 $$

Where A is the absorbance of ABTS and B is the absorbance of sample.

### Anticholinesterase assays

Acetyl cholinesterase (AChE) and butyrylcholineaterase (BChE) were used to examine the enzyme inhibitory potential of crude extract and sub-fractions using Ell men’s assay [19] which is based on the hydrolysis of Acetylthiocholine iodide or butyrylthiocholine iodide by respective enzyme and the formation of 5-thio-2-nitrobenzoate anion followed by DTNB complexation to give yellow color compound which is detected after reaction time of 15 min by spectrophotometer.

#### Preparation of required solutions

Phosphate buffer was prepared by dissolving 13.6 g/L of KH_2_PO_4_ and 17.4 g/L of K_2_HPO_4_, separately in 100 ml of distilled water. The phosphate buffer of pH 8 was prepared by mixing 6% of KH_2_PO_4_ with 94% of K_2_HPO_4_. The AChE and BChE were diluted in freshly prepared buffer of pH 8.0 to achieve a final concentration of 0.03 and 0.01 U/mL respectively. To prepare DTNB solution (0.0002 M) in 10 ml of phosphate buffer and shake well and then the final volume was made up to 100 ml. The AChEI and BChEI solutions (0.0005 M) were also prepared in phosphate buffer separately in 100 ml.

#### Spectroscopic analysis

For spectroscopic determination of the enzyme inhibition, 1 ml of crude extract and sub fractions (125–1000 μg/ml) were taken separately in a series of test tubes. Then to these solutions 100 μl of enzyme and DTNB solutions were added and kept in incubator for 15 min at 25 °C. After this 100 μl substrates (AChEI, BChEI) were added to each test tube and allowed to stand for 15 min to proceed the reaction. Then the absorbance was measured at 412 nm. For negative control solution all the above mentioned components except plant extracts were mixed in the mentioned order. Galanthamine was used as a positive control. The same procedure mentioned above was used for reaction mixture of positive control and absorbance was measured at 412 nm. For each sample absorption was recorded for 4 min. Percent activity of enzyme and percent inhibition were calculated using following relation:

3 $$ V=\frac{\Delta  Abs}{\Delta  T} $$

4 $$ \% enzyme\ activity=\frac{V}{Vmax}\times 100 $$

5 $$ \%\mathrm{enzyme}\ \mathrm{inhibition}=100-\%\mathrm{enzyme}\ \mathrm{activity} $$

Where V represents the rate of reaction in the presence of inhibitor and V_max_ is the rate of reaction without inhibitor.

### Determination of total phenolic content

The total phenolic contents were estimated using Follin-ciocalteu assay [20]. For the preparation of required solution of these extracts (crude and sub fractions) 5 mg of each samples were dissolved in 5 ml of methanol. After this 1 ml of Follin-ciocalteu reagent was taken and added into 10 ml of distilled water to prepared diluted solution of Follin-ciocalteu reagent. For the preparation of reaction mixtures 1 ml of extracts solutions were taken in a test tube and 9 ml of distilled water were added to each. Then 1 ml of diluted solution of Follin-ciocalteu reagent was also added to each test tube and allowed to stand for 6 min. After 6 min 10 ml of 7% of sodium carbonate and 10 ml of distilled water was added to the reaction mixture. Then the mixture was diluted further to 25 ml with the addition of distilled water and were shaken thoroughly. The absorbance of each solution was recorded after 90 min at 760 nm through UV spectrophotometer. For the determination of total phenolic content standard gallic acid curve (0 to 100 mg/ml) was drawn. The total phenolic contents were expressed as milligrams of Gallic acid equivalent (mg GAE/g per gram of dry sample).

### Determination of total flavonoid content

For the determination of total flavonoid contents, the procedure of Park et al. was followed [21]. About 5 mg of each of these extracts (crude and sub fractions) were dissolved in 5 ml of methanol. From which 1 ml was taken from each and mixed with 9 ml of distilled water. Then to the mixture 1 ml of 5% sodium nitrate (NaNO_2_) was added and allowed to stand for 6 min. Then 2 ml of 10% aluminum chloride solution was added to it and allowed to stand for 5 min. After this 2 ml solution of 1 M sodium hydroxide was added sequentially to each tube. The absorbance of this reaction mixture was recorded at 510 nm through UV spectrophotometer. For the determination of total flavonoids standard quercetin solution curve (0 to 200 mg/ ml) was constructed. The total flavonoid contents were expressed in term of Quercetin equivalent (mg.QE/g of dry sample).

### Determination of phenolic contents through HPLC

The HPLC system used was an Agilent 1260 infinity High-performance Liquid chromatography (HPLC) system having basic parts like quaternary pump, auto sampler, degasser and ultra violet (UV) detector. The separation was achieved via Agilent Zorbax Eclipse C18 column. The gradient system comprises of solvent A (methanol; acetic acid: deionized water, 10:2:88, *v*/v and solvent B (methanol: acetic acid deionized water, 90: 2: 8, *v*/v). the efficient gradient program was started with 100% A at 0 min, 85% A at 5 min, 50% A at 20 min, 30% A at 25 min, and 100% B from 30 to 40 min. The flow rate of sample was 1 ml/min. The identification was performed by using retention times, available standards and UV spectra. Quantification of the identified compounds was on the basis of % peaks.

For the preparation of samples for HPLC analysis, 1 g of the powder sample (in case of fractions solid extract) was dissolved in 10 mL methanol/water (5, 5) mixture, overtaxing for 15 min and then shaking for 1 h. After this the mixtures were filter with Whattman filter paper having pore size of 0.7 μm. Then filtered samples were centrifuged at 4000 rpm for 15 min. After centrifugation the solvent was evaporated under vacuum at 35 °C up to 2 ml volume. It was filtered again through PTFE Agilent with pore size of 0.45 μm. The filtrate were collected into 2 ml HPLC vials and were labeled with proper code and placed in a refrigerator. Standard solutions of quercetin, morin, rutin, mandelic acid, pyrogallol, hydroxy benzoic acid, phloroglucinol and chlorogenic acid were prepared in methanol. A standard chromatogram was developed for the mixture of HPLC standards mentioned above. HPLC chromatogram of *Aesculus indica* fruit crude extract and chloroform, ethyl acetate, n-hexane and aqueous sub fractions were also obtained.

By comparing these chromatograms with the standard one, the phenolic compounds identified in the crude extract were; quercetin, phloroglucinol, mandelic acid and hydroxy benzoic acid. In the ethyl acetate fraction the phenolic compounds identified were; quercetin, mandelic acid and hydroxy benzoic acid. In the other fraction (n-hexane, chloroform, and aqueous) the amount of phenolic compound present were very small as compared to crude extract and ethyl acetate fraction.

### Isolation of phenolic compounds

The HPLC chromatograms obtained for the fractions showed that ethyl acetate fraction contains greater number of phenolic compounds and was therefore subjected to silica gel column for the isolation of bioactive compounds.

Column chromatography is generally used to isolate and purify the components of complex mixtures. Silica gel column chromatography was used for separation and isolation of pure compounds from ethyl acetate fraction. For the preparation of column, silica gel was suspended in required solvent and left for 4 h to soak and was then poured into the column. The solid ethyl acetate extract fraction was dissolved in the minimum amount of particular solvent in which it was soluble and filtered to remove impurities, which could otherwise will cause diffusion problems while packing the column. After this, the slurry of the fractions with silica gel were developed which was then loaded over silica gel column using a pipette with the help glass rods with great care in such a way to not disturb the top of the column. After loading the sample to column it was eluted with ethyl acetate and n-hexane mixtures {1:4 (20%), 1:3 (25%), 3:7 (30%), 7:13 (35%), 2:3 (40%), 9:11 (45%), 1:1 50%)} with the help of peristaltic pump (SEKO, Italy). The vials collected were spotted on TLC and visualized in the UV light.

The 12 sub fractions were obtained and were again subjected to column chromatography (pencil column). Two compounds quercetin and mandelic acid were isolated in pure form. The purity and isolation of the compounds were confirmed through HPLC where a single broad peak was observed. The structure of these compounds was elucidated by spectrophotometric techniques like FT-IR and H^1^NMR.

### Estimation of IC_50_ values

Concentrations of the plant extract at which 50% of inhibition was observed (IC_50_), were calculated amongst the inhibition percentages against the extract concentrations via the Excel program.

### Statistical data analysis

All the assays were repeated in triplicate and values have been expressed as means ±standard error means (SEM). Statistical analyses were performed by Two-way analysis of variance (ANOVA), followed by Bonferroni post-tests. The difference was considered to be statistically significant when the *p* value was less than 0.05.

### Determination of R^2^ and Y

Regression (y) and linear correlation (R^2^) for phenolic and flavonoid contents vs various activities (anticholinesterase and antioxidants) were determined using Microsoft Excel 2007.

## Additional file

Additional file 1: **Figure S1.** % DPPH free radical scavenging effect of *Aesculus indica* crude extract and their sub fraction along with ascorbic acid taken as a standard. **Figure S2.** Percent ABTS scavenging effect of *Aesculus indica* of crude extract and their sub fraction along with ascorbic acid at various concentrations. **Figure S3.** % AChE inhibition potential of *Aesculus indica* fruit crude extract and their different sub fraction. **Figure S4.** % BChE inhibition potential of *Aesculus indica* fruit crude extract and their different sub fraction. **Figure S5.** Total phenolic content in Aesculus indica fruit crude extract and different sub fraction. **Figure S6.** Total Flavonoid content in Aesculus indica fruit crude extract and different sub fraction. **Figure S7.** FTIR Spectra of the isolated compound Quercetin. **Figure S8.** NMR Spectra of Quercetin. **Figure S9.** FT-IR spectra of the isolated compound Mandalic acid. **Figure S10.** NMR Spectra of Mandalic acid. **Figure S11.** % DPPH free radical scavenging effect of quercetin and mandalic acid isolated from Aesculus *indica* fruit using ascorbic acid taken as a standard. **Figure S12.** % ABTS free radical scavenging effect of quercetin and mandalic acid isolated from Aesculus *indica* fruit using ascorbic acid taken as a standard. (DOCX 645 kb)
