# Supplementary material for: Isolation of quercetin and mandelic acid from Aesculus indica fruit and their biological activities
Source: BMC Biochem. 2018 Jun 26;19:5. doi: 10.1186/s12858-018-0095-7 (PMC6019818; doi:10.1186/s12858-018-0095-7)
Supplement: Supplementary file 1 — Figure S1. % DPPH free radical scavenging effect of Aesculus indica crude extract and their sub fraction along with ascorbic acid taken as a standard. Figure S2. Percent ABTS scavenging effect of Aesculus indica of crude extract and their sub fraction along with ascorbic acid at various concentrations. Figure S3. % AChE inhibition potential of Aesculus indica fruit crude extract and their different sub fraction. Figure S4. % BChE inhibition potential of Aesculus indica fruit crude extract and their different sub fraction. Figure S5. Total phenolic content in Aesculus indica fruit crude extract and different sub fraction. Figure S6. Total Flavonoid content in Aesculus indica fruit crude extract and different sub fraction. Figure S7. FTIR Spectra of the isolated compound Quercetin. Figure S8. NMR Spectra of Quercetin. Figure S9. FT-IR spectra of the isolated compound Mandalic acid. Figure S10. NMR Spectra of Mandalic acid. Figure S11. % DPPH free radical scavenging effect of quercetin and mandalic acid isolated from Aesculus indica fruit using ascorbic acid taken as a standard. Figure S12. % ABTS free radical scavenging effect of quercetin and mandalic acid isolated from Aesculus indica fruit using ascorbic acid taken as a standard. (DOCX 645 kb) [file 12858_2018_95_MOESM1_ESM.docx]

**Additional file 1**

**Figure S1:** % DPPH free radical scavenging effect of *Aesculus indica* crude extract and their sub fraction along with ascorbic acid taken as a standard

**Figure S2:** Percent ABTS scavenging effect of *Aesculus indica* of crude extract and their sub fraction along with ascorbic acid at various concentrations.

**Figure S3:** % AChE inhibition potential of *Aesculus indica* fruit crude extract and their different sub fraction

**Figure S4:** % BChE inhibition potential of *Aesculus indica* fruit crude extract and their different sub fraction

**Figure S5**: Total phenolic content in Aesculus indica fruit crude extract and different sub fraction

**Figure S6**: Total Flavonoid content in Aesculus indica fruit crude extract and different sub fraction


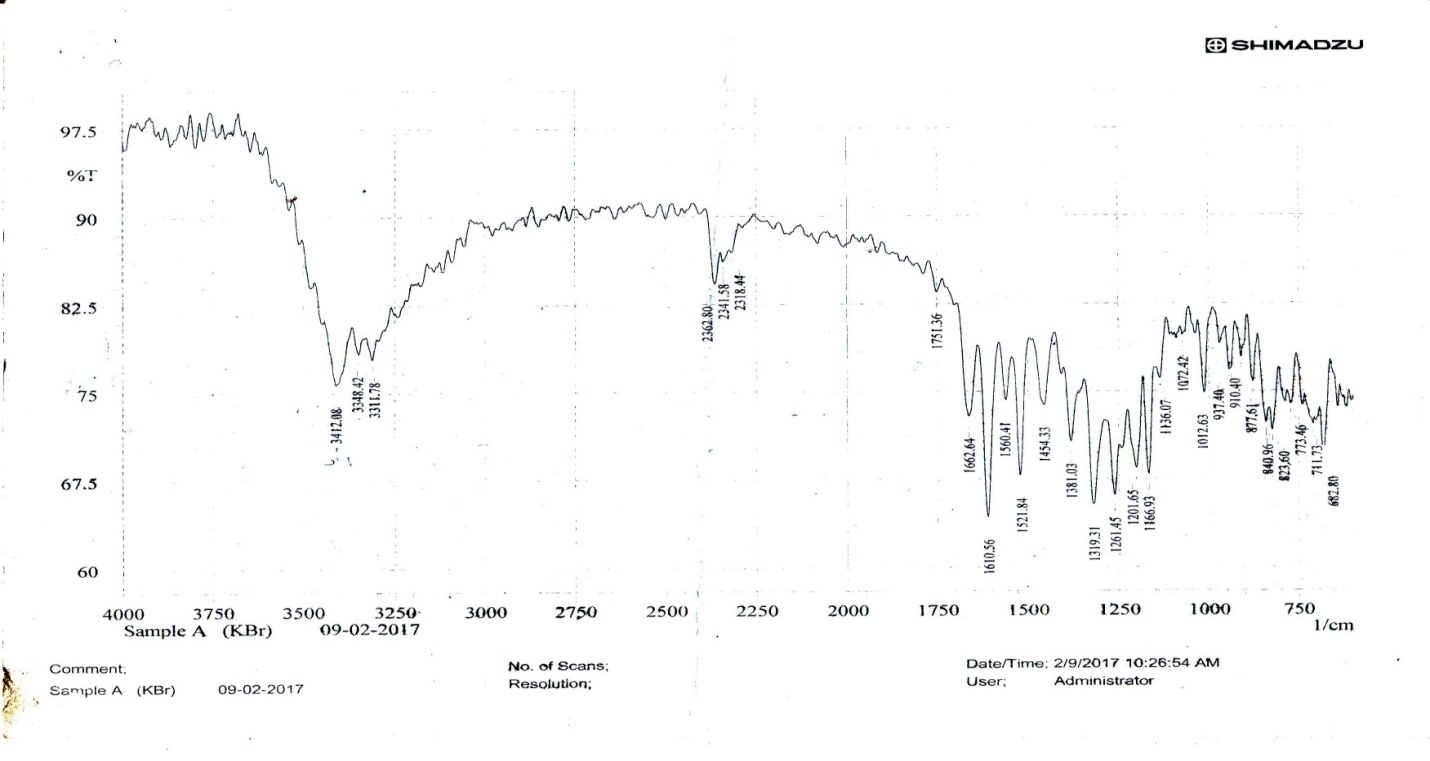


**Figure S7:** FTIR Spectra of the isolated compound Quercetin


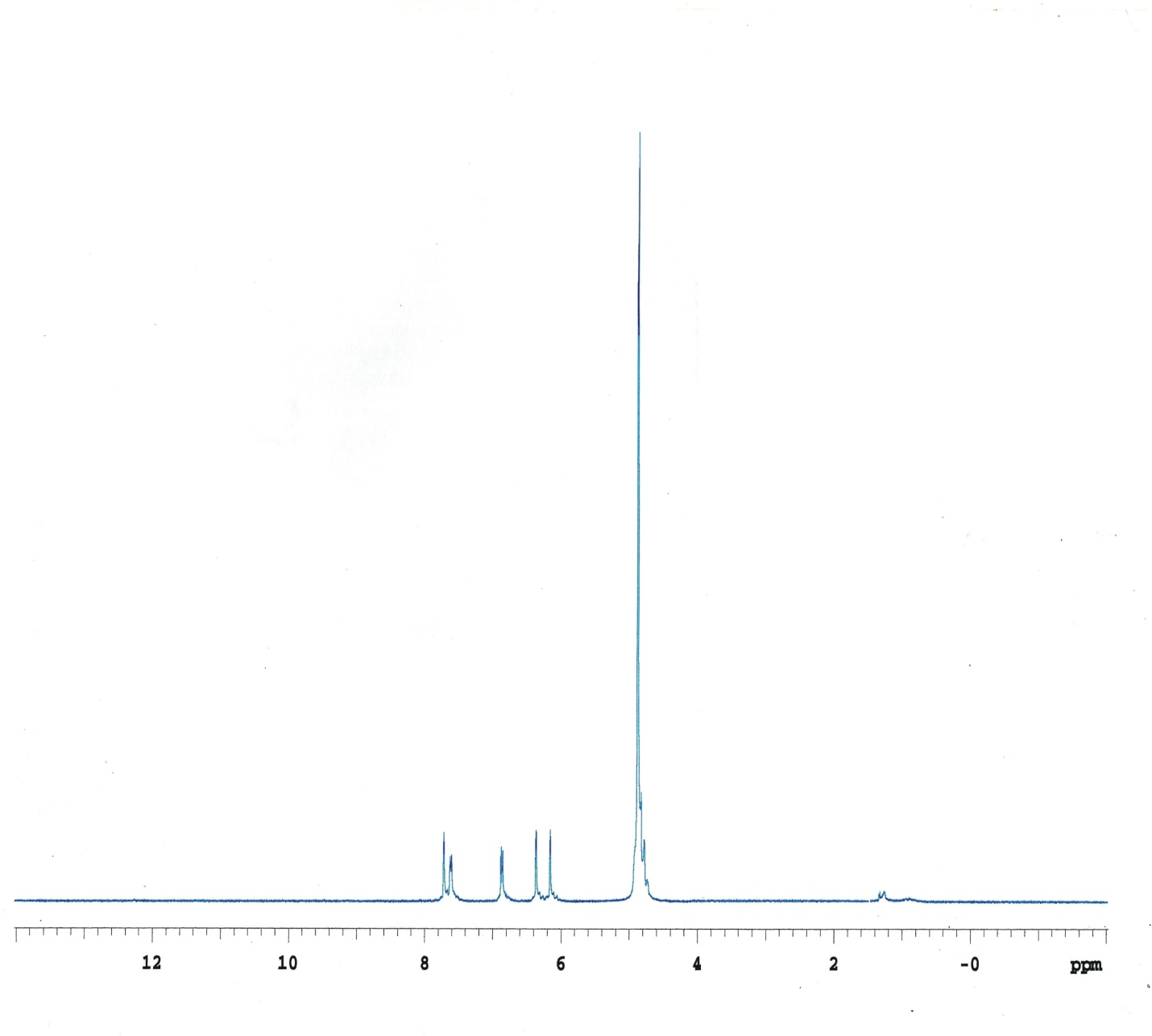


**Figure S8**: NMR Spectra of Quercetin


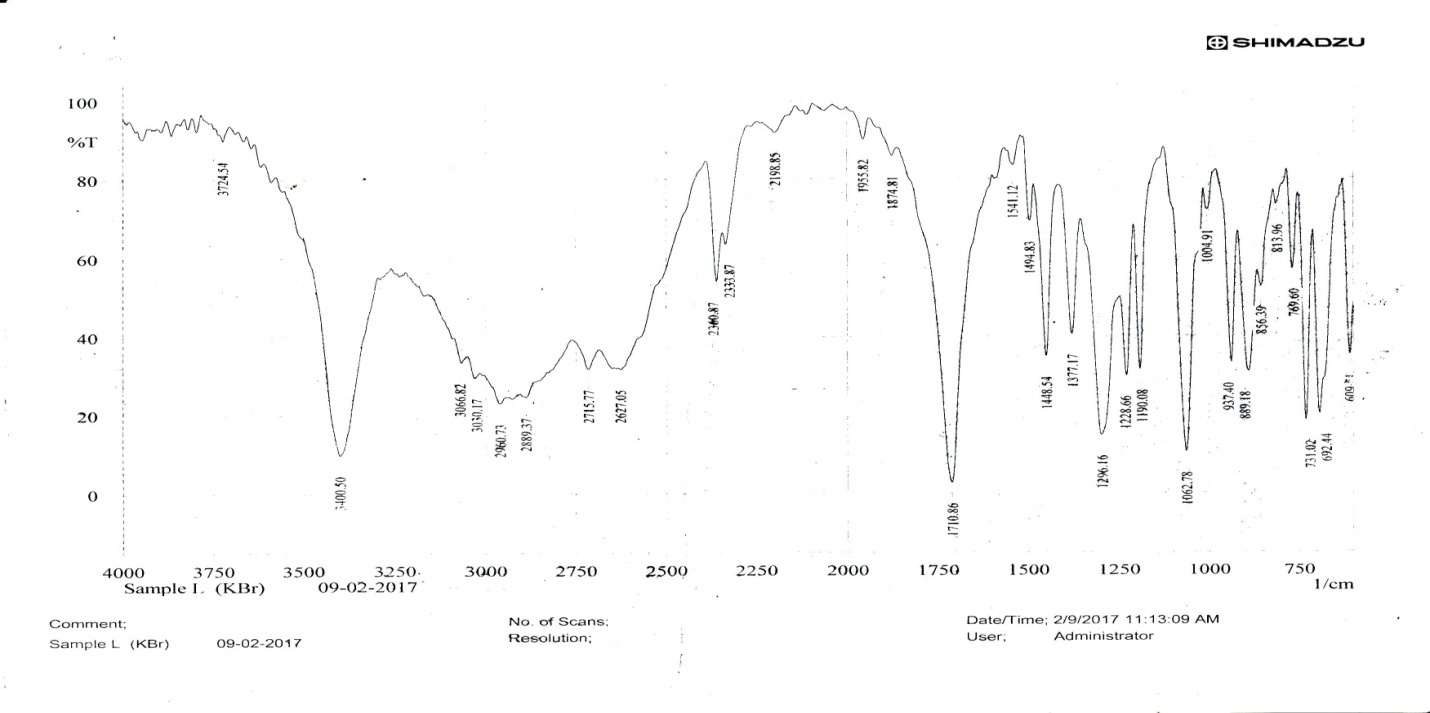
 **Figure S9:** FT-IR spectra of the isolated compound Mandalic acid


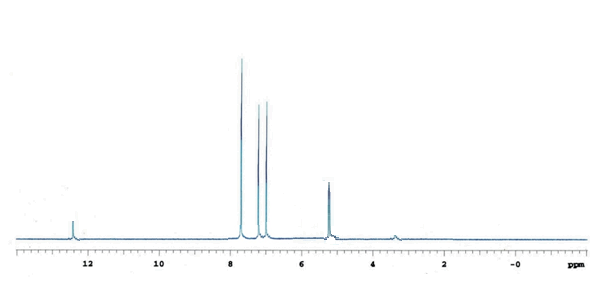


**Figure S10**: NMR Spectra of Mandalic acid

**Figure S11:** % DPPH free radical scavenging effect of quercetin and mandalic acid isolated from Aesculus *indica* fruit using ascorbic acid taken as a standard

**Figure S12:** % ABTS free radical scavenging effect of quercetin and mandalic acid isolated from Aesculus *indica* fruit using ascorbic acid taken as a standard
